# Acceptance, Usability and Health Applications of Virtual Worlds by Older Adults: A Feasibility Study

**DOI:** 10.2196/resprot.5423

**Published:** 2016-06-02

**Authors:** Nicole Cook, Sandra L Winkler

**Affiliations:** ^1^ Master of Public Health program College of Osteopathic Medicine Nova Southeastern University Fort Lauderdale, FL United States; ^2^ James A. Haley Veterans Hospital Tampa, FL United States

**Keywords:** Second Life, virtual worlds, older adults, seniors, health care, training

## Abstract

**Background:**

Virtual worlds allow users to communicate and interact across various environments, scenarios, and platforms. Virtual worlds present opportunities in health care to reduce the burden of illness and disability by supporting education, rehabilitation, self-management, and social networking. The application of virtual worlds to older adults who bear the burden and cost of health conditions associated with age has not been evaluated.

**Objective:**

The aim of this study is to explore the usability, ease of use, and enjoyment of a virtual world by older adults, the types of virtual world activities that older adults may engage in, and the perceptions of older adults regarding the application of virtual worlds in health care.

**Methods:**

This quasi-experimental pre-post design research was guided by the Technology Acceptance Model (TAM). Participants were recruited from a Lifelong Learning Institute (LLI) program at Nova Southeastern University. Participants attended four training sessions over a 5-week period in the Second Life (SL) virtual world. Subjects were surveyed before and after the training on perceived ease of use, attitudes towards technology, behavioral intention to use the system, facilitating conditions, effort expectancy, and self-efficacy.

**Results:**

Older adults (N=19) completed the informed consent and attended the first training session, and 11 participants (58%, 11/19) completed the full training and the post survey. Completers (82%, 9/11) were more likely than non-completers (37%, 3/8) to consider themselves technologically savvy (*P*=.048), and to express confidence in being able to use the virtual world (100%, 11/11 vs 37%, 3/8; *P*=.002). All completers (100%, 11/11) perceived that SL has application in health behaviors and disease and reducing social isolation among people who are homebound. Of the completers, 10 (91%, 10/11) responded that they enjoyed learning how to use SL. Completers suggested that future trainings include more assistants and smaller groups.

**Conclusions:**

This pilot study suggests that virtual worlds can be both a feasible and an applicable method to promote health among some seniors. Future research on virtual worlds with older populations should consider using state-of-the art technology including large monitors, providing a minimum of one trainer for every two to three participants, and distributing a comprehensive training manual at the start of the training to support organization and recall.

## Introduction

As the population ages, the burden and cost of health conditions associated with age, including chronic disease and dementing illnesses is growing [1]. Strategies that promote self-management of chronic disease, cognitive function, and social connectedness contribute to improved health outcomes and quality of life [2-4]. Among other methods, computer-based interventions including computer-based gaming, interactive multimedia systems, exergaming, and virtual worlds are emerging as innovative methods to promote health among older adults [5-8].

Virtual worlds are three-dimensional computer-simulated environments that users navigate via Avatars to communicate and interact across various environments, scenarios, and platforms. In the past decade, virtual worlds have emerged as important tools for socialization, engagement, networking, and entertainment, virtually changing how people interact, communicate, and collaborate [9]. Internet-based virtual worlds offer opportunities in health care to reduce the burden of illnesses and disability through promoting rehabilitation [10-12], self-management of disease [13-18], healthy lifestyle behaviors [19-21], and social networking [11,22]. However, most research and evaluations on the application of virtual worlds in health are conducted among younger, computer literate populations, though the application to older adults has been identified [23].

Older adults comprise an important population for virtual computer and Web-based health interventions, including virtual worlds [24]. Today, 59% of adults 65 years of age use the Internet [24]. Usage increases with higher education, higher income, and lower age. Among seniors who reported using the Internet, more than two thirds of them go online every day. Similarly, among seniors who use social networking sites, 81% reported using them daily or near-daily.

Use of virtual worlds among seniors is low, but is expected to increase [25,26]. Currently there is a lack of evidence regarding the acceptance, adoption, and health applications of virtual worlds among older adults. Here, a pilot study was conducted to inform on the feasibility and potential health care applications of using a virtual world environment with older adults. The specific research aims of this study are as follows: (1) to understand the usability, ease of use, and enjoyment of a virtual world environment by older adults, (2) probe engagement and types of virtual world activities by older adults, and (3) identify older adults’ perceptions regarding the application of virtual worlds in health care.

## Methods

### Study Design

**Figure 1:**
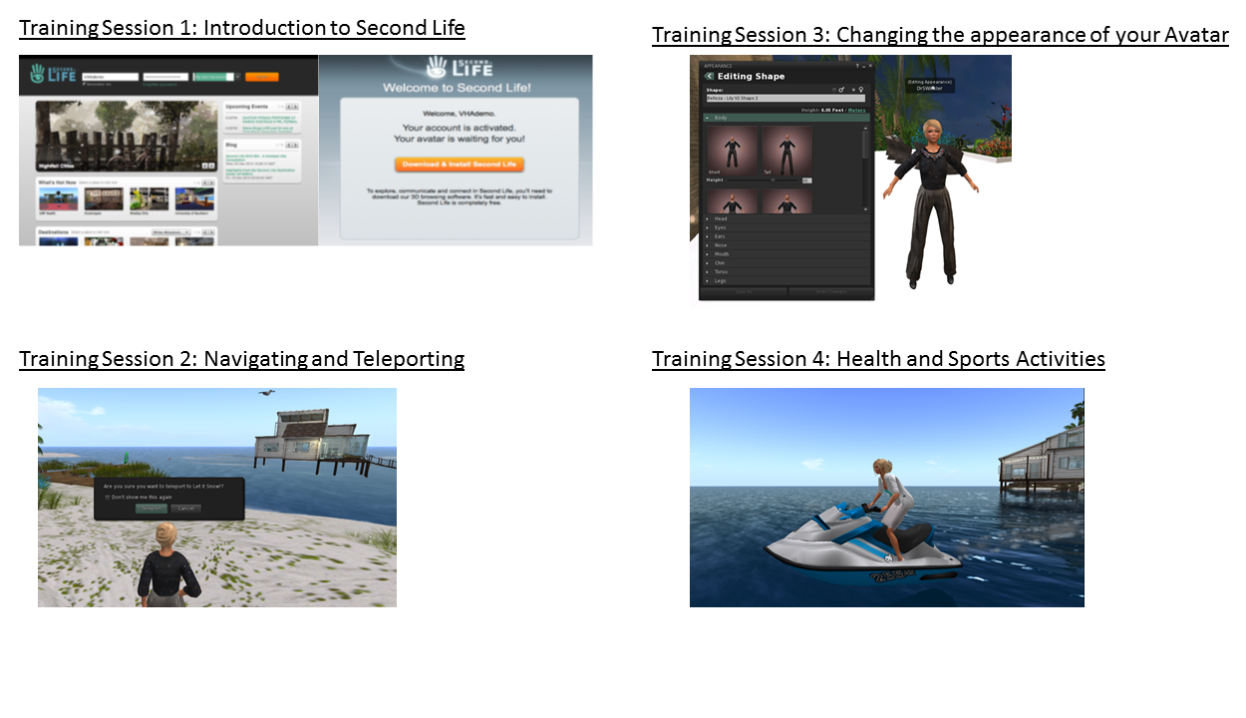
Screen shots demonstrating examples of activities performed in Second Life during training sessions.

A quasi-experimental pre-post design was used. After providing written consent subjects completed a pre-test, which included questions regarding demographics, computer and digital device usage, and potential advantages of virtual worlds (Multimedia Appendix 1). Subjects then attended four training sessions over a 5-week period using the Second Life (SL) virtual world in the computer lab at Nova Southeastern University. SL is the largest virtual world. Conceptualized by Philip Rosedale who founded Linden Lab, SL offers a persistent, open, unlimited, and highly customizable space. The content of SL is created by its users who rent virtual land (islands). The training was conducted by a faculty member. Another faculty member and one student were available to provide direct support to participants at each training session. The training topics covered were (1) introduction to SL, "friending" and messaging (week 1); (2) navigating and teleporting in SL (week 2); (3) changing the appearance of your Avatars (week 3); and (4) health and sports activities (week 4). Screen shots demonstrating examples of activities performed in SL during training are shown in Figure 1. Subjects completed a 19 question post-test assessing their experience with the virtual world, the potential advantages of virtual worlds, and the specific health applications of virtual worlds. The post-test is provided in Multimedia Appendix 2. Subjects who completed all four training sessions received a US $60 gift card to a local supermarket chain.

### Participants and Setting

Participants were recruited from the Lifelong Learning Institute (LLI) at Nova Southeastern University’s College of Osteopathic Medicine. As an educational extension program the LLI attracts many active and educated older adults from the nearby community.

Study investigators made recruiting announcements during LLI events and were available during LLI educational program breaks to discuss the training program with interested members. Though recruitment was slow at first, an interested LLI board member agreed to make several announcements to his peers, appealing to those interested in technology resulting in higher interest. Inclusion criteria for the study included having an email address and availability to attend the training programs. There were no exclusion criteria.

### Conceptual Framework

The research was guided by the Technology Acceptance Model (TAM) [27]. The TAM is an extension of the Theory of Reasoned Action (TRA) which predicts a person’s behavioral intention based on attitudes and subjective norms [28]. TAM extends TRA by predicting acceptance of information technologies and assumes that two major variables of interest drive an individual’s acceptance of technology: perceived usefulness and perceived ease of use [29]. TAM has been used successfully to test a variety of computer technologies in dividers settings and user groups, including in health care [30-35]. TAM evolved over several iterations into the Unified Theory of Acceptance and Use of Technology (UTAUT) [36]. To our knowledge, this is the first application of TAM and the extended UTAUT towards understanding acceptance and adoption of virtual worlds among older adults.

### Outcome Measures

The pre- and post-survey instruments were developed by the investigators based on validated items used in the development and testing of the TAM and UTAUT [27,36]. The tool measured perceived ease of use, attitudes towards technology, behavioral intention to use the system, facilitating conditions, effort expectancy, and self-efficacy. The pre-survey included questions on demographic characteristics, current computer and device usage, technological savviness, confidence in using the virtual world, and perceived usefulness of virtual worlds. The post-survey tool included the same set of questions regarding perceived usefulness as in the baseline survey and assessed activities performed in SL as a result of the training. The post-survey tool also included five open-ended questions to inform on specific lessons that could be learned through this pilot and applied to future research on virtual worlds and seniors.

### Data Collection

Study data were collected and managed using Research Electronic Data Capture (REDCap) electronic data capture tools hosted at Nova Southeastern University [37]. REDCap is a secure, Web-based application designed to support data capture for research studies providing (1) an intuitive interface for validated data entry; (2) audit trails for tracking data manipulation and export procedures; (3) automated export procedures for seamless data downloads to common statistical packages; and (4) procedures for importing data from external sources.

### Statistical Analyses

We performed bivariate analysis of baseline completer and non-completer demographic characteristics using *t* test for independent samples for age and the Pearson chi-square statistic for categorical data. Paired-sample *t* tests were used to compare changes in self-reported usefulness on a scale from 1 (strongly agree) to 5 (strongly disagree) among completers and to estimate power for future studies. An alpha of .05 was used for each test without correction for multiple comparisons. Descriptive statistics were used to describe activities completed and perceptions regarding the application of virtual worlds to health. All analyses were conducted in SPSS (V23).

## Results

### Study Participants

There were 19 older adults who completed the informed consent and baseline survey and 10 older adults who completed the 4-week training program. One participant missed the last class, however, the material was reviewed with the participant at the end of the prior training. This participant’s post-test was included with the completers, for an overall number of 11 completers, and a completer to non-completer rate of 58% (11/19).

The demographic and baseline characteristics of completers and non-completers are summarized in Table 1. The mean age of participants was 71.2 among both completers and non-completers. Completers (82%, 8/11) were statistically more likely than non-completers (37%, 3/8) to consider themselves technologically savvy (*P*=.048) and to express confidence in being able to use the virtual world (100%, 11/11 vs 37%, 3/8, *P*=.002). There was an absolute difference in mean hours on the computer of 3.65, but this difference was not significant. Though not significant, completers were more likely to agree or strongly agree with the perceived benefits of a virtual world in terms of usefulness in managing health (36%, 4/11 vs 25%, 2/8), usefulness for social interaction (45%, 5/11 vs 37%, 3/8), usefulness to themselves (36%, 4/11 vs 25%, 2/8), and usefulness in terms of improving quality of life (27%, 3/11 vs 25%, 2/8).

### Activities Performed in Second Life

Activities performed in SL by completers are listed in Table 2. While faculty and graduate students verified that all completers performed all of the activities assessed at each class, several participants in the post-survey said that they were not able to "friend each other", find new places to visit, go shopping in SL or change their Avatar’s appearance in SL.

**Table 1:** Baseline demographic and characteristics of non-completers and completers of a virtual world training program (N=19).

Characteristic	Non-completers (n=8)	Completers (n=11)	*P* value
Age^a^, mean	71.2	71.2	.99
Hours on computer per week^a^, mean	8.75	12.4	.31
Male, %	50	36	.55
College graduate, %	75	91	.55
"I consider myself to be technologically savvy", agree/strongly agree (%)	37	82	.048
"I am confident I will be able to use the virtual world", yes (%)	37	100	.002
"A virtual world could be useful in managing my health", agree/strongly agree (%)	25	36	.60
“A virtual world could be useful for social interaction", agree/strongly agree, %	37	45	.73
"A virtual world could be useful to me", agree/strongly agree (%)	25	36	.60
"Using a virtual world could improve the quality of my life", agree/strongly agree (%)	25	27	.91

^a^One completer did not provide age or mean hours on computer per week.

**Table 2:** Activities in Second Life performed by completers during the training program (N=11).

Activity	n, (%)
“Friending each other”	6 (54)
Finding new places to visit	9 (82)
Teleporting	11 (100)
Shopping	8 (73)
Changing your Avatar’s appearance	9 (82)
Sports activities (eg, jet skiing, parasailing, fishing, etc)	11 (100)

### Usefulness of Second Life

The change in perceived usefulness of SL was analyzed in terms of managing health, social interaction, overall usefulness, and improvement in quality of life among completers (Table 3). Though only the usefulness of SL for social interaction approached significance (*P*=.046) there was improvement across three of the four questions from baseline to follow-up.

### Application of Second Life to Health and Ease of Use

The application of SL to health and the ease of use of SL among completers was assessed (Table 4). All subjects (100%, 11/11) responded that SL is applicable to health behaviors and disease and that SL can be applied to reduce social isolation among people who are homebound. Of the participants, 10 (91%, 10/11) responded that they enjoyed learning how to use SL. In addition, 8 (73%, 8/11) and 9 (82%, 9/11) responded that it was easy for them to learn to create their Avatar and that it was easy for them to navigate their Avatar in SL, respectively.

**Table 3:** Changes in mean score of usefulness of Second Life among completers improved from baseline to follow-up (N=11).

Usefulness	Baseline completers score^a^, mean	Follow-up completers score^a^, mean	*P* value
"A virtual world could be useful in managing my health"	2.82	2.27	.051
"A virtual world could be useful for social interaction"	2.55	1.91	.046
"A virtual world could be useful to me"	2.60	2.40	.55
"Using a virtual world could improve the quality of my life"	2.80	3.10	.47

^a^Mean score based on a 1 (strongly agree) to 5 (strongly disagree) scale.

**Table 4:** Participants' perception regarding ease of use and applications of Second Life to health (N=11).

Application	n (%)
Application of Second Life to health behaviors and disease	11 (100)
Application of Second Life to self-management and chronic conditions	6 (54)
Application of Second Life to support brain health among people with dementing illnesses	7 (64)
Application of Second Life to support behavior and lifestyle change (eg, physical activity)	6 (54)
Application of Second Life to reduce social isolation among people who are homebound	11 (100)
I enjoyed learning how to use Second Life?	10 (91)
It was easy for me to learn to create an Avatar in Second Life?	8 (73)
It was easy for me to learn to navigate my Avatar in Second Life?	9 (82)

### Qualitative Feedback on Second Life

Participants also responded to open-ended questions regarding what they liked about SL, what they least liked about SL, their motivation to participate in the training, and potential improvements both in terms of the SL application and the training program. In summary, respondents liked the graphics, the variety of virtual experiences (eg, virtual travel) and engaging in fantasies and other sports activities that they would not normally engage in real life (eg, flying, power sailing). Several respondents also said they enjoyed designing their own Avatars and creating environments that are "outside the norm". In terms of what they liked least, several respondents said they found SL frustrating (difficulty maneuvering the Avatar and/or not knowing where to go when they arrive at a site), that the on-screen help is limited and hard to understand, and that there is a long learning curve. One respondent also noted that the computer lab environment where the training took place was overloaded and crashed. With regards to motivation to participate, all participants responded that they wanted to help with interesting research and/or to learn something new. Feedback with regards to improving the usability of SL for the senior population and improving the training are provided in Table 5. The responses to both questions are combined as there was significant overlap.

**Table 5:** Respondent feedback on improving the usability of Second Life for the senior population and improving the training program for seniors in SL (N=11).

Feedback	n (%)
Provide more assistants during training; provide individualized or small-group training	5 (45)
Teach exercises slower in a step-by-step manner	4 (36)
Provide a comprehensive, more organized training program and manual; simplify directions	3 (27)
Enlarge the print on the screen	2 (18)
Make it less challenging to navigate in Second Life	1 (9)
Provide the training on an Apple platform	1 (9)

## Discussion

### Principal Findings

Findings from this pilot study to investigate the feasibility and potential health care applications of using a virtual world environment with older adults suggest that perceived usefulness of virtual worlds in terms of health, managing health, overall usefulness, and improving quality of life increased following the training program. Seniors who considered themselves technologically savvy and are confident in their ability to learn the virtual world technology were more likely to complete the training compared to those who had less confidence and self-reported themselves as not technologically savvy. Seniors who completed the training were successful at creating Avatars, learning to "friend each other", instant messaging, teleporting, and engaging in activities in SL. All completers reported that SL has application in health and managing disease, as well as to reduce social isolation among people who are homebound.

### Limitations

A limitation of the study was the high number of drop-outs. During the first class participants (N=19) were instructed to set up their SL account. However, the trainers did not know that there was a limit on the number of new accounts that could be set up per Internet Protocol (IP) address. As a result, many participants had difficulty creating accounts. Trainers resolved this issue by connecting to personal hotspots on cell phones, but the interruption contributed to frustration that may have resulted in drop-outs. Participants were also frustrated that they had to wait, often for several minutes, for assistance. In retrospect, because subjects learned how to use an Avatar at different rates and had varying levels of attention, several smaller groups should have been formed with at least one trainer to two to three participants. Persons providing assistance to participants needed to have mastered basic Avatar and SL skills, which was not always the case in this study. While most completers reported they enjoyed learning SL (100%, 11/11), that they found it easy to create an Avatar (73%, 8/11), and navigate in SL (82%, 9/11), there were other important lessons learned through this pilot. Participants experienced frustration with using and navigating in SL. Some of the frustration can be attributed to the computer lab conditions. The computer lab was equipped with laptops, which met the recommended requirements for screen resolution and graphics, but only had 14 inch screens. In addition, some activities in SL were slow at certain points in the training, likely due to Internet congestion caused by a large number of computers working simultaneously with intense graphic displays. A few of the computers even crashed.

In addition to increasing the number of trainers available to support setting up accounts and using high-end technology with large monitors, suggested improvements for future trainings include providing more support during each of the trainings and providing a comprehensive training manual. There were several challenges to maintaining a tempo acceptable for all participants. First, some participants failed to remember their username and password in subsequent trainings and then not having access to their email accounts to access the email from SL to reset their credentials. Second, subjects were at different stages of readiness to use an Avatar in a virtual world, making it difficult to learn new skills as a group. More SL technologically savvy students wanted to press ahead with learning new skills while others in the class were still learning previously-taught tasks. Future projects may want to consider grouping participants by tempo. In addition, while handouts were provided to students at the beginning of each training, there was no overall comprehensive training manual. In retrospect, such a manual would have helped with organizing the material and facilitating recall of previously taught activities. Plus, many participants wanted to practice on their home computers for which the manual would have been conducive.

### Effect Size

One of the purposes of a pilot study is to calculate an effect size for more robust, larger-scale studies. To this end, in a post-hoc analysis, we calculated the magnitude of the effect of the change in participant reported usefulness (composite of four questions) of SL pre- and post-training. Using a power of 80% and an alpha of .05, we calculated that 90 participants should be enrolled in future studies to achieve a medium effect size (30%). Precaution should be taken when applying our effect size to future studies as we modified TAM questions to fit our population and virtual world technology. It is also important to note that older adults who participated in this pilot had a college degree and that estimated results may vary if the study is replicated with a less-educated population.

### Conclusions

The purpose of this pilot study was to test the feasibility and applicability of virtual worlds to older adults. This pilot suggests that virtual worlds can be both a feasible and applicable method to promote health among some seniors. Future research on virtual worlds with older populations should consider using state-of-the art technology including large monitors, providing a minimum of one trainer for every two to three participants, and distributing a comprehensive training manual at the start of the training to support organization and recall.

## Appendix

Multimedia Appendix 1 Pre-test questionnaire to assess acceptance, usability, and health applications of virtual worlds among older adults.

Multimedia Appendix 2 Post-test assessing the experience with the virtual world, potential advantages of virtual worlds, and specific health applications of virtual worlds among completers.

## Abbreviations

LLI: Lifelong Learning Institute
REDCap: Research Electronic Data Capture
SL: Second Life
TAM: Technology Acceptance Model
TRA: Theory of Reasoned Action
UTAUT: Unified Theory of Acceptance and Use of Technology
