# Supplementary material for: Acceptance, Usability and Health Applications of Virtual Worlds by Older Adults: A Feasibility Study
Source: JMIR Res Protoc. 2016 Jun 2;5(2):e81. doi: 10.2196/resprot.5423 (PMC4911513; doi:10.2196/resprot.5423)
Supplement: Multimedia Appendix 1 [file resprot_v5i2e81_app1.pdf]

Demographic information and computer experience survey

Please complete the survey below.

Thank you!

1 What is your email? ________________________________

2 What is your gender?
- ◯ Female
- ◯ Male

3 What is your age? ________________________________

4 What is your highest level of education?
- ◯ Did not graduate high school
- ◯ High school graduate
- ◯ College graduate
- ◯ Post-graduate degree

5 Which devices or equipment do you use on a regular basis (>3 times per week)? Check all that apply:
- ☐ Smart Phone (ex. Iphone or other cell phone with touch screen)
- ☐ Tablet (ex. Ipad)
- ☐ Personal Computer
- ☐ Laptop
- ☐ Digital Camera
- ☐ E-reader (ex. Kindle, Nook)
- ☐ video games
- ☐ Other

6 How many hours do you spend on a computer each week? ________________________________

7 Which websites or software applications do you use on a regular basis (>3 times per week) check all that apply?
- ☐ Email
- ☐ Facebook
- ☐ Twitter
- ☐ Instagram/Snapchat/ other photo sharing program
- ☐ Facetime
- ☐ You Tube
- ☐ Gaming
- ☐ News
- ☐ Travel websites
- ☐ Health websites
- ☐ Google sites
- ☐ Other

8 Do you consider yourself to be technologically savvy?
- ◯ Yes
- ◯ No

9 I am confident I will be able to use the virtual world
- ◯ Strongly Agree
- ◯ Agree
- ◯ Undecided
- ◯ Disagree
- ◯ Strongly Disagree

10 Have you ever navigated an avatar through a Virtual World?
- ◯ Yes
- ◯ No
- ◯ Not sure

11 Are you familiar with Virtual World (such as Second Life)?
- ◯ Yes
- ◯ No

12 A virtual world could be useful in managing my health
- ◯ Strongly Agree
- ◯ Agree
- ◯ Undecided
- ◯ Disagree
- ◯ Strongly Disagree

13 A virtual world could be useful for social interaction

- ◯ Strongly Agree
- ◯ Agree
- ◯ Undecided
- ◯ Disagree
- ◯ Strongly Disagree

14 A virtual world could be useful to me

- ◯ Strongly Agree
- ◯ Agree
- ◯ Undecided
- ◯ Disagree
- ◯ Strongly Disagree

15 Using a virtual world could improve the quality of my life

- ◯ Strongly Agree
- ◯ Agree
- ◯ Undecided
- ◯ Disagree
- ◯ Strongly Disagree
