# Supplementary material for: Acceptance, Usability and Health Applications of Virtual Worlds by Older Adults: A Feasibility Study
Source: JMIR Res Protoc. 2016 Jun 2;5(2):e81. doi: 10.2196/resprot.5423 (PMC4911513; doi:10.2196/resprot.5423)
Supplement: Multimedia Appendix 2 [file resprot_v5i2e81_app2.pdf]

Second Life Post-Program Survey

Please complete the survey below.

Thank you!

1 What is your email? __________________________________

2 Are you familiar with Virtual World (such as Second Life)?
- ◯ Yes
- ◯ No

3 A virtual world could be useful in managing my health
- ◯ Strongly Agree
- ◯ Agree
- ◯ Undecided
- ◯ Disagree
- ◯ Strongly Disagree

4 A virtual world could be useful for social interaction
- ◯ Strongly Agree
- ◯ Agree
- ◯ Undecided
- ◯ Disagree
- ◯ Strongly Disagree

5 A virtual world could be useful to me
- ◯ Strongly Agree
- ◯ Agree
- ◯ Undecided
- ◯ Disagree
- ◯ Strongly Disagree

6 Using a virtual world could improve the quality of my life
- ◯ Strongly Agree
- ◯ Agree
- ◯ Undecided
- ◯ Disagree
- ◯ Strongly Disagree

7 I enjoyed learning how to use Second Life?
- ◯ Strongly Agree
- ◯ Agree
- ◯ Undecided
- ◯ Disagree
- ◯ Strongly Disagree

8 It was easy for me to learn to create an Avatar in Second Life?
- ◯ Strongly Agree
- ◯ Agree
- ◯ Undecided
- ◯ Disagree
- ◯ Strongly Disagree

9 It was easy for me to learn to navigate my Avatar in Second Life?
- ◯ Strongly Agree
- ◯ Agree
- ◯ Undecided
- ◯ Disagree
- ◯ Strongly Disagree

10 Which components of Second Life did you use? (Check all that apply)
- ☐ Instant Messaging
- ☐ Finding Friends
- ☐ Finding new places to visit
- ☐ Teleporting
- ☐ Shopping
- ☐ Changing your Avatar's appearance
- ☐ Sport activities (jet skiing, parasailing, fishing, etc)

11 Are you likely to continue to use Second Life after this class?
- ◯ Yes
- ◯ No
- ◯ Don't know/not sure

12 What do you think are the potential applications of Second Life in health care?

- ☐ education of health behaviors and diseases
- ☐ self-management of chronic conditions
- ☐ support brain health among people with dementing illnesses
- ☐ support behavior and lifestyle change (for example, physical activity)
- ☐ reduce social isolation among people who are homebound

13 Please indicate any other potential applications you think there are for Second Life that are not mentioned above.

____

14 Please share one to three things you most liked about Second Life?

____

15 Please share one to three things you least liked about Second Life?

____

16 What motivated you to come to this class and participate for 4 weeks?

____

17 How can the usability of Second Life be improved for the senior population?

____

18 How can we improve the training we conducted on Second Life?

____

19 If timing and convenience allowed, would you be interested in being a trainer to work with new Second Life users on another project?

- ◯ yes
- ◯ no
- ◯ maybe

20 Thank you for participating. We will have a $60 Target gift card for you ready on Monday 11/24. How would you like to receive it? (Choices: can leave with Heike Doss in Lifelong Learning Institute or Mail it to my house address- please provide address)

____
